# Anticipating depression trajectories by measuring plasticity and change through symptom network dynamics

**DOI:** 10.1192/j.eurpsy.2025.10083

**Published:** 2025-08-15

**Authors:** Claudia Delli Colli, Aurelia Viglione, Alessandro Giuliani, Igor Branchi

**Affiliations:** 1 Center for Behavioral Sciences and Mental Health, Istituto Superiore di Sanità, Rome, Italy; 2 Environment and Health Department, Istituto Superiore di Sanità, Rome, Italy; 3 Institute of Advanced Studies, University of Amsterdam, Amsterdam, The Netherlands

**Keywords:** connectivity, mental health, network analysis, precision psychiatry, prediction, recovery, remission, treatment response

## Abstract

**Background:**

Network analysis is a promising approach for elucidating the dynamics of the transition from psychopathology to well-being. Recently, symptom connectivity strength has been proposed as a measure of plasticity – the capacity to change disease severity. Yet, empirical findings remain inconsistent. We propose that this inconsistency can be resolved by recognizing that the interpretation of connectivity strength varies along the recovery process from depression, whether at baseline or during clinical change.

**Methods:**

We analyzed 2,710 depressed patients from the STAR*D dataset, grouped by the magnitude of change in depressive score. Symptom network connectivity was estimated from QIDS-C items at three time points: (i) baseline, (ii) change – defined as when clinical change in depression score occurs, (iii) post-change - corresponding to when the maximum clinical change is achieved.

**Results:**

At baseline, connectivity strength predicts the maximum clinical change, inversely correlating with its magnitude (*ρ* = −0.95, *p* = 0.001). At the change time point, connectivity strength parallels clinical change (*ρ* = 0.92, *p* = 0.002). A direct and significant association between connectivity strength and depression severity emerges only at the change (ρ = 0.98, p = 0.0003) and post-change (ρ = 0.95, p = 0.001) time points.

**Conclusions:**

The interpretation of connectivity strength for predicting depression trajectories varies by timepoint: at baseline, it measures plasticity -- the capacity for change -- whereas during clinical change, it indicates the magnitude of change in symptom severity. This framework supports the reliability of this prognostic marker for designing personalized therapeutic interventions in psychiatry.

## Introduction

Plasticity is defined as the capacity for change, and thus the potential to modify brain functioning and mental states [1]. It is increasingly recognized as a critical factor in determining recovery trajectories in mental illness as it underlies the reorganization of neural and mental processes during the transition from psychopathology to well-being [2, 3]. It is noteworthy that the definition of plasticity implies that it is neither inherently beneficial nor detrimental, as it affects the likelihood of a transition without setting its direction. The direction is set by contextual factors, such as living conditions or the subjective appraisal of the quality of life [3, 4]. Indeed, a growing body of evidence increasingly shows that treatments that enhance plasticity produce context-dependent effects, amplifying the influence of contextual factors in shaping mental health and recovery trajectories [5–11]. Consequently, the outcome of different levels of plasticity has to be interpreted in the light of context [1, 12].

Recently, within the network theory of mental health – which conceptualizes psychiatric disorders as complex, dynamic systems of interconnected psychological features (e.g., symptoms) [13–15] – a novel network-based approach to measuring plasticity has been proposed [2, 16] and subsequently validated [8, 17, 18]. Plasticity has been operationalized as the inverse of symptom connectivity strength – defined as the degree to which psychological features co-occur and, therefore, are connected to each other [16]. Consequently, stronger connectivity indicates lower plasticity and vice versa. Clinical studies showed that weaker network connectivity strength at baseline is associated with faster recovery from major depressive disorder and positive treatment response [8, 17, 19–22]. However, these findings have not been consistently replicated, as other studies reported no association [20, 23–25], thereby casting doubts on the potential clinical applicability of the network-based approach to mental disorders.

To account for these discrepancies, we propose that connectivity strength should be differently interpreted depending on the specific time point at which it is measured along the transition to recovery, whether at baseline or during the period of clinical change in depression severity. Specifically, we hypothesized that, at baseline – when no consistent change in depression score is occurring as no therapeutic intervention has yet been implemented – connectivity strength predicts plasticity and, therefore, an individual’s capacity for future modifications in disease severity [2]. In contrast, once the change in depressive score is underway, connectivity strength is directly associated with – and therefore measures – the magnitude of the ongoing change. Such an association is expected because coordination among the elements of any system is required for a coherent system shift from one state to another [26].

To validate our hypothesis, we conducted a secondary analysis of the Sequenced Treatment Alternatives to Relieve Depression (STAR*D) dataset. From the original trial sample [27], we selected 2,710 patients with depression who met the inclusion criteria. Depression scores were analyzed at baseline and at weeks 4, 6, 9, and 12 over a 12-week period of Level 1 of the clinical trial to identify the interval during which each patient exhibited the maximum clinical change. Symptom network connectivity, assessed using the Quick Inventory of Depressive Symptomatology (QIDS)-C items, was estimated at three time points: (i) at baseline, corresponding to enrollment; (ii) at the change time point, that is the period of clinical change in depression score, which marks a phase of ongoing transformation potentially leading to recovery; and (iii) at the post-change time point, which coincides with the attainment of the maximum clinical change in depression score. We expected that, at baseline, connectivity strength predicts the magnitude of clinical change achieved over the course of the trial, in line with the view that lower baseline connectivity anticipates a higher potential for modification and vice versa. By contrast, once the change in depression severity is underway, we expected connectivity strength to directly correlate with the extent of that change, thereby serving as an indicator of its magnitude.

## Methods

### Overall design

The research presented here complies with all relevant ethical regulations. We conducted a secondary analysis on the dataset of the STAR*D study (ClinicalTrials.gov Identifier: NCT00021528). The original study was approved and monitored by the institutional review boards at each of the 14 participating institutions, a national coordinating center, a data coordinating center, and the data safety and monitoring board at the National Mental Health Institute, National Institutes of Health, USA. All participants involved in the original study provided written informed consent at the beginning of the study.

### Participants

The STAR*D is a randomized clinical trial of outpatients with major depressive disorder designed to prospectively evaluate the effectiveness of pharmacological and psychotherapeutic treatment as described in previous studies [28]. Briefly, the STAR*D enrolled a total of 4,040 outpatients (18–75 years old) with nonpsychotic depression (17-item Hamilton Depression Rating Scale score ≥ 14). Only data concerning Level 1 of the clinical trial were considered in the present analysis. Patients were excluded if they (i) were pregnant or breastfeeding; (ii) had a primary diagnosis of bipolar, psychotic, obsessive-compulsive, or eating disorders; (iii) had general medical conditions contraindicating the use of protocol medications; (iv) had substance dependence; or (v) had a clear history of nonresponse or intolerance. The STAR*D protocol involved clinical visits at 4, 6, 9, and 12 weeks.

### Depressive symptoms

Depressive symptoms were collected using the clinic version of the QIDS-C, consisting of 16 clinician-rated items measuring the nine criterion symptom domains that define Major Depressive Disorder according to the Diagnostic and Statistical Manual of Mental Disorders, 4th Edition, Text Revision. The scores for three domains – sleep, appetite/weight, and restlessness/agitation – are based upon the maximum score of two or more questions. The remaining domains are each assessed using a single item. As a result, the original 16 items are consolidated into 9 symptom domains. All nine domains are scored from 0 (i.e., no problem) to 3 points (i.e., severe problem). The overall total score is calculated by summing the scores of all domains, and it ranges from 0 (i.e., not depressed) to 27 (i.e., most depressed).

### Context

The Quality-of-Life Enjoyment and Satisfaction Questionnaire-short form (Q-LES-Q-SF) was employed to evaluate the context at baseline. The Q-LES-Q-SF is a self-reported questionnaire, with 16 items, derived from the general activities scale of the original 93-item form. The questionnaire is adopted in clinical practice to measure the degree of enjoyment and satisfaction experienced by patients in various areas of their daily life (e.g., family, work, and daily activities). Each item is scored on a scale from 1 (i.e., very poor) to 5 points (i.e., very good). Two of the 16 items refer to general aspects of life and are not included in the calculation of the overall total score, which thus ranges from 14 (i.e., very poor) to 70 (i.e., very good). Based on the median, we identify patients experiencing a poor context (Q-LES-Q-SF score < 45) and those experiencing a good context (Q-LES-Q-SF score ≥ 45).

### Outcomes

As a primary outcome, we calculated the differences between the QIDS-C scores at each clinical visit (from weeks 4–12) and the QIDS-C at baseline (i.e., 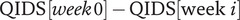 , where 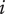 corresponds to each clinical visit week). Among these five values, we identified the maximum one in terms of absolute value (i.e., ΔQIDS), representing the maximum clinical change in depressive symptoms achieved by each participant over the course of 12 weeks, either improvement or worsening. We defined the time point immediately preceding the one at which the maximum clinical change is attained as the change phase, and the time point coinciding with the attainment of the maximum clinical change in depression score as the post-change phase. For example, if a subject achieves the maximum clinical change at week 6, the change phase would correspond to week 4, and the post-change phase to week 6 (Supplementary Table 1). We split the population into eight groups, each representing a range of two units in ΔQIDS (Table 1). Due to the limited number of patients with ΔQIDS >16 (*n* = 185), we combined these participants with those who had ΔQIDS equal to 16 (*n* = 86) into a single group. Within each group, we calculated the average ΔQIDS (i.e., mean of maximum clinical change) as the primary outcome. As a secondary outcome, we assessed the change in QIDS-C from baseline to the last week of the level 1 (i.e., 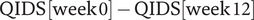 ).

**Table 1.** Group characteristics

Maximum clinical change (ΔQIDS)	Sample size	Connectivity strength (baseline)	ΔQIDS (mean ± SD)
[0–3]	322	3.52	2.14 ± 0.88
[4–5]	276	3.40	4.51 ± 0.50
[6–7]	380	2.99	6.56 ± 0.50
[8–9]	398	2.96	8.52 ± 0.50
[10–11]	462	2.63	10.50 ± 0.50
[12–13]	329	2.72	12.53 ± 0.50
[14–15]	273	2.18	14.46 ± 0.50
[16–24]	271	2.38	17.65 ± 1.72

Abbreviations: QIDS, Quick Inventory of Depressive Symptomatology; SD, standard deviation.

Finally, to focus on a more clinically relevant outcome, we calculated both symptom improvement ( 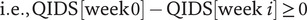 , where 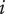 corresponds to each clinical visit week) and clinical response (i.e., a 50% reduction in symptoms relative to baseline). Specifically, we replicated the main analysis (see section below), including only subjects whose maximum change reflected an improvement in symptoms. Additionally, we compared subjects who consistently achieved a clinical response at any of the available weeks to those who did not.

### Statistical analysis

All statistical analyses were performed in R version 4.2.3. From the original sample size enrolled in the STAR*D, we have included 2,710 patients with available information (Supplementary Figure 1).

### Difference in sample characteristics

To assess the difference in age and depression severity at baseline, a one-way analysis of variance (ANOVA) was applied using the aov function. Post-hoc comparisons were performed using the TukeyHSD function. For repeated measurements, post-hoc pairwise comparisons were conducted using two-tailed paired *t*-tests with Bonferroni correction for multiple comparisons, via the pairwise.t.test function.

### Network analysis

Networks were estimated using the estimateNetwork function in the bootnet R package. Symptom networks consist of nodes (i.e., nine depressive symptoms domains derived from QIDS-C) and edges. Following standard methodology in the psychometric literature [29], we estimated the network using a Gaussian Graphical Model (GGM), in which edges represent conditional pairwise associations between symptoms, controlling for all other symptoms in the network. Due to the ordinal nature of the symptoms – measured on a Likert scale – the partial correlation matrix was estimated using Spearman’s rank correlation. Global network connectivity strength, defined as the sum of the absolute weights of all edges, was calculated for each network. For the eight groups described in the *Outcome* section, we estimated the networks at the following time points: baseline, change, and post-change. All networks were estimated cross-sectionally, as longitudinal data were not available. To ensure robustness, we repeated the analysis employing the Least Absolute Shrinkage and Selection Operator (LASSO) algorithm, which forces the small partial correlation coefficients to zero and produces a sparse network structure [30, 31]. To address potential bias in the analysis, which may arise from the outcome and network estimation relying on the same scale (i.e., QIDS-C), we replicated the analysis using the self-reported version of the QIDS (QIDS-SR16). Because connectivity strength was computed across multiple time points (baseline, change, and post-change), we were constrained to using QIDS-C/SR16, as the 17-item Hamilton Depression Rating Scale was only administered at baseline. Finally, since depressive symptoms are measured on an ordinal scale and, thus, are neither continuous nor normally distributed, we conducted a sensitivity analysis by estimating a full correlation matrix using a nonparametric approach that, unlike GGM models, does not assume multivariate normality.

### Network comparison test (NTC)

Networks were compared based on global network connectivity strength – defined as the sum of the magnitude of the weighted connections – using the NTC function in the Network Comparison Test (Version: 2.2.2) R package. NTC is a permutation-based test that randomly regroups participants from the network repeatedly [30]. The resulting distribution under the null hypothesis (i.e., assuming both groups are equal) was used to test the observed difference between the networks (i.e., whether the observed statistics fall within the 95th percentile for a significance level of 0.05). We compared baseline global network connectivity strength between responders and nonresponders.

### Spearman’s rank correlation

The Spearman’s rank correlation coefficient rho (*ρ*) and its relative *p*-values were computed using the cor and cor.test functions from the stats R package.

## Results

### Sample characteristics

A total of 2,710 subjects – corresponding to 67% of the original sample included in the STAR*D – were included in the analysis due to the availability of QIDS-C data at the change time point, which were necessary to estimate ΔQIDS (Supplementary Figure 1). A total of 2,572 subjects included in our analysis showed overall positive ΔQIDS values, reflecting an improvement over the weeks. The remaining 138 subjects (4%) showed a negative ΔQIDS reflecting a worsening in depression severity. Among them, data on depressive symptoms are available only for 60 subjects (44%). Of these, after the worsening phase, 13 persist in their condition, showing no change in depressive symptoms, 20 showed a modest improvement of 1–2 points, 25 showed a moderate improvement of 3–7 points, one subject improved by 10 points, and another one by 15 points. The included 2,710 subjects were divided into eight groups based on ΔQIDS (Table 1), as described in the *Methods* section. These groups differed significantly in baseline QIDS-C scores (F[7,2702] = 78.21, *p* < 0.001; Supplementary Figure 2). Specifically, post-hoc comparisons revealed that groups with higher ΔQIDS (i.e., [12–13], [14–15], and [16–24]) exhibited significantly higher baseline QIDS-C scores compared to the other groups (Tukey’s post-hoc: *p* < 0.01). Interestingly, the group with the higher QIDS-C score (indicating greater symptom severity) displays lower baseline connectivity strength compared to the other groups. Additionally, the groups differed in terms of age (F[7,2701] = 4.35, *p* < 0.0001; Supplementary Figure 2), with the group showing the highest ΔQIDS (i.e., [16–24]) being significantly older than the other groups (Tukey’s post-hoc: *p* < 0.01). Finally, the female-to-male ratio shows a similar distribution in all groups, with a consistently higher percentage of female participants in each group.

### The maximum clinical change achieved is proportional to the connectivity at baseline

**Figure 1.**
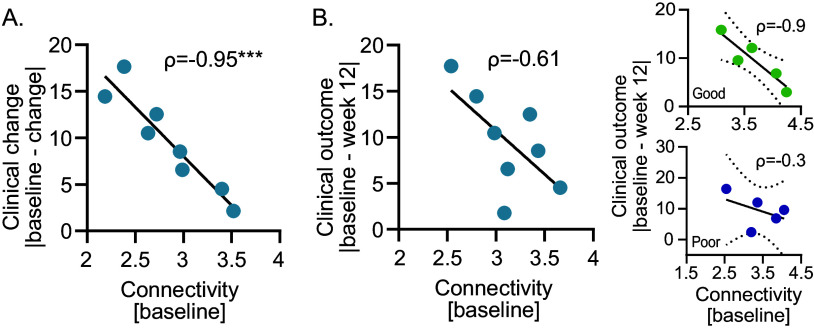
Connectivity strength is inversely correlated to (A) maximum clinical change achieved across the weeks and (B) change achieved by week 12. Spearman’s rank correlation between connectivity strength estimated at baseline using the QIDS-C and the ΔQIDS (averaged within each group), calculated at (A) the week of maximum change and (B) week 12. A two-sided Spearman rank correlation test was used to estimate the correlation. ρ, Spearman coefficient, ***p = 0.001. Sample sizes are described in Table 1. Insets on the right show correlations between change achieved at week 12 and connectivity strength at baseline for two representative subgroups: green dots indicate individuals in a good context, while blue dots represent those in a poor context. Black dot line: 95% confidence bands of the best-fit line.

Baseline connectivity strength significantly correlates with the maximum clinical change achieved across the week (Spearman’s rank correlation rho (*ρ*) = −0.95, *p* = 0.001; Figure 1A and Supplementary Figures 4A and 5). When considering only symptom improvement (i.e., including only subjects whose maximum change reflected improvement), we found similar results (*ρ* = −0.95, *p* = 0.001; Supplementary Figure 2A). Furthermore, when analyzing clinical response, we observed that patients who consistently responded across the weeks exhibited lower baseline connectivity compared to those who did not respond (NTC test *p* < 0.05). This finding suggests that baseline network connectivity predicts the maximum extent of potential clinical change, with lower connectivity associated with a higher potential for change. As expected, we found that network connectivity strength was positively correlated with QIDS-C items variance (*ρ* = 0.78, *p* = 0.02; data not shown), indicating that lower variance across items was linked to lower connectivity.

### The clinical outcome achieved at the end of the trial is defined by plasticity through context interplay

Although not statistically significant, baseline connectivity strength shows a moderate correlation with the symptom changes observed by week 12 (*ρ* = −0.61, *p* = 0.11; Figure 1B) and the improvement (*ρ* = −0.66, *p* = 0.08; Supplementary Figure 2B). The predictive value of connectivity strength for week 12 outcomes (i.e., clinical outcome) became more evident when patients were stratified by perceived context, distinguishing between those who reported a poor versus a good context. Specifically, network connectivity strongly correlates with symptom change at week 12 only in patients who perceived the context as good (*ρ* = −0.90, *p* = 0.08), but not in those who perceived it as poor (*ρ* = −0.30, *p* = 0.68). Given the limited sample size, we interpret the correlation coefficients primarily as indicators of effect size, rather than focusing solely on statistical significance. Additionally, we compared the slopes of the correlations between baseline connectivity strength and both the largest clinical change in depression score achieved at any point during the clinical trial (Figure 1A) and clinical outcome (Figure 1B) reveals the former to be stronger and significantly different from the latter (Fisher-Z = −2.7, *p* = 0.02).

### The connectivity strength parallels the clinical change

**Figure 2.**
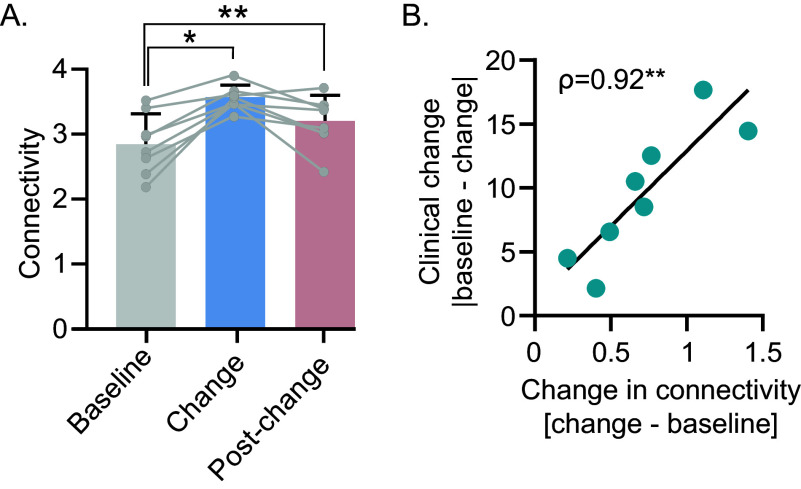
Change in connectivity strength predicts the maximum clinical change achieved across the weeks. (A) Connectivity strength increases from baseline during the change phase. Two-tailed paired t-tests with Bonferroni correction: *p = 0.03, **p = 0.003. (B) Correlation between the change in connectivity strength from baseline to change phase and the maximum clinical change (i.e., ΔQIDS averaged within each group). A two-sided Spearman rank correlation test was used to estimate the correlation. *ρ*, Spearman coefficient, **p = 0.002.

To assess whether connectivity strength differed across time points, a repeated-measures ANOVA was performed, revealing a significant main effect of time (F[2, 14] = 17.29, *p* < 0.05, Figure 2A). Bonferroni-corrected pairwise comparisons revealed significant differences in connectivity between the following timepoint baseline versus change (*p* = 0.03) and baseline versus post-change (*p* = 0.003), but not between change and post-change (*p* = 0.07). When considering only clinical improvement, we observed similar results (Supplementary Figure 2C). These results suggest that connectivity increases in parallel with ongoing changes in depressive symptoms. Additionally, the difference in network connectivity strength measured at baseline and at the change phase correlates with maximum change (*ρ* = 0.92, *p* = 0.002; Figure 2B and Supplementary Figure 4B) and improvement (*ρ* = 0.92, *p* = 0.002; Supplementary Figure 2D), indicating that greater changes in connectivity were associated with greater symptom improvement. When estimating the networks using a nonparametric approach, we obtained overlapping results (see Supplementary Figure 6).

### Baseline connectivity strength and depression severity

**Figure 3.**
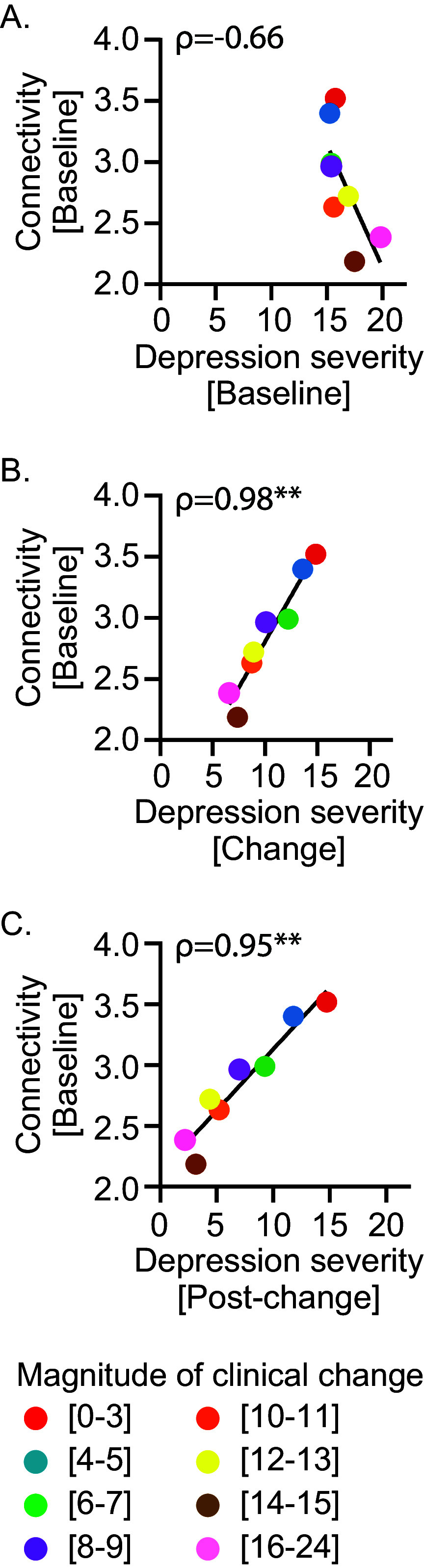
Connectivity strength and depression severity across the different timepoints: baseline, change, and post-change. Correlation between connectivity strength at baseline and depression severity measured with QIDS-C (A) at baseline, (B) at change phase, and (C) post-change. A two-sided Spearman rank correlation test was used to estimate the correlation. *ρ*, Spearman coefficient, **p = 0.007.

When examining the relationship between baseline connectivity strength and depression severity, we found that the association varied depending on the time point at which severity was assessed. Specifically, at baseline, the correlation between connectivity strength and disease severity measured at the same time point is not significant (*ρ* = −0.66, *p* = 0.08; Figure 3A). By contrast, it emerges at the change time point (*ρ* = 0.98, *p* = 0.0004; Figure 3B) and at the post-change time point (*ρ* = 0.95, *p* = 0.001, Figure 3C).

## Discussions

The present results indicate that the interpretation of connectivity strength among symptoms for predicting depression trajectories varies along the recovery process. At baseline, connectivity is a measure of plasticity and, thus, predicts the magnitude of future clinical change in depression score a patient will achieve over time, with weaker connectivity associated with larger change and vice versa. Conversely, once the clinical change is underway, connectivity strength corresponds to the magnitude of the ongoing change. A direct and significant correlation between baseline connectivity strength and disease severity is not present at baseline but emerges only at the change and post-change time points.

The inverse correlations between baseline connectivity strength and both the largest change in depression score achieved at any point during the clinical trial (Figure 1A) and final clinical outcome at week 12 (Figure 1B) align with previous studies reporting densely connected symptom networks to be associated with poorer prospects for transitioning from psychopathology to mental well-being [8, 17, 19–22]. In addition, the comparison between these two correlations reveals the former to be stronger and significantly different from the latter. This difference further supports the interpretation of connectivity strength assessed at baseline as a measure of capacity for change and, thus, of plasticity [2, 16]. The weaker correlation between baseline connectivity strength and clinical outcome emerges because recovery depends not only on plasticity but also on its interplay with context, as plasticity promotes improvement only when paired with favorable contextual conditions [3, 8]. Accordingly, the correlation between baseline connectivity strength and clinical outcome was found to be strong in a good but nearly absent in a poor quality of context (Figure 1B inset).

Upon entering the change phase – marked by consistent depressive score change either toward improvement or worsening – connectivity strength increases compared to baseline (Figure 2A). This increase is strongly correlated with the magnitude of clinical change, further highlighting the association between these two processes (Figure 2B). This result aligns with previous findings reporting stronger connectivity within the symptom network in patients experiencing a reduction in depressive symptoms following either antidepressant treatment [20, 32–34] or psychotherapy [22, 35, 36], compared to those showing a persistent symptomatology. Other studies that investigated connectivity strength at the individual level within the network of affect states also confirmed that stronger connectivity during the change phase is associated with larger shifts in depression severity [37, 38].

The increase in connectivity strength observed during the change time point has been attributed to several factors, including greater variability in symptoms [38, 39] and the concurrent modifications of multiple symptoms contributing to the overall improvement of the symptomatology [35]. We propose that such an increase in connectivity may also reflect the growing coordination occurring among the elements of any system to achieve transitions across states, with the degree of their coordination directly related to the extent of transition [26]. This view is in line with previous findings showing that the probability of, and the temporal proximity to, an upcoming shift between a depressed and healthy state is associated with an increase in correlation among emotions or affect states [37, 40–43]. Notably, this increase concerns not only autocorrelations but also correlations among distinct variables within the same system [44–46]. This phenomenon has been shown across disciplines, from physics to finance [47].

The correlations between connectivity strength at baseline and the severity of depressive symptomatology at the three transition time points – baseline, change, and post-change – are consistent with the view of baseline connectivity strength as a measure of plasticity and not of depression severity. Indeed, baseline connectivity strength inversely correlates with depression severity at baseline (Figure 3A). In addition, as individuals with weaker connectivity at baseline show greater change in depression severity across the time points, a strong and direct correlation between baseline connectivity strength and disease severity emerges only at the change and post-change time points (Figure 3B,C). This is because, while the change phase is underway, individuals with high plasticity are capable of achieving greater symptom amelioration, whereas those with low plasticity, due to their limited capacity for change, persist in pathological conditions. This results in a gradient where more plastic individuals exhibit less severe symptomatology. Importantly, these findings reconcile our interpretation of baseline connectivity strength as a measure of plasticity with alternative interpretations that consider it as a potential index of depression severity [48, 49].

Previous studies have yielded inconsistent findings regarding the hypothesis that connectivity among symptoms can serve as a predictive marker of transition in mental health. Some studies linked weak connectivity to an increased likelihood of transition [17, 19, 21, 22], while others reported no significant association [20, 23–25]. Our findings – demonstrating that the interpretation of connectivity strength and, consequently, its predictive value varies across the transition process – may reconcile these inconsistencies. Therefore, data used to predict mental health trajectories must be collected at clearly identified timepoints – either at baseline, before the initiation of the therapeutic intervention, or while the clinical change is underway. Otherwise, the lack of information regarding whether patients involved were in the baseline or had already entered the change phase at the time of assessment may lead to inconclusive results.

The present findings hold the potential to significantly inform the development of strategies aimed at predicting individual disease trajectories. The ability to measure either the capacity for change or the magnitude of change – based on analyses of symptom networks at different time points during therapeutic intervention – could significantly improve the identification of such trajectories in depression, a key challenge that currently limits the timely delivery of personalized interventions. This approach may help overcome the limited reliability of currently available markers [50, 51] and the reliance on a “trial and error” strategy for evaluating treatment efficacy, a practice that not only delays the establishment of therapeutic benefit but also increases the risk of adverse side effects and suicide [50, 52].

Our study has limitations that should be acknowledged. First, network analyses were conducted at the group level because multiple symptom assessments for each patient were not available in STAR*D. Therefore, further investigation is warranted to explore the association between connectivity strength and clinical change at the individual level based on longitudinal data. Nevertheless, existing studies that explored network connectivity at the individual level [37, 38, 41, 43] have reported findings that are consistent with the framework proposed here. In addition, the role of the treatment regimen was not considered in the analysis because the limited sample size precluded a statistical investigation of its relationship with connectivity strength. Nevertheless, this limitation does not imply that the treatment regimen is unimportant [53].

In conclusion, there is an urgent need to identify effective markers that can optimize personalized treatments [54], ultimately leading to a reduction of the burden of depression and mental illness not only at clinical but also at societal and economic levels [55, 56]. Without accurate prediction of disease trajectories, therapeutic strategies might be prematurely discontinued or altered while they remain clinically beneficial, potentially resulting in loss of patient compliance or dropout. The assessment of connectivity strength within the symptom network at different time points along the transition process yields clinically meaningful predictive insights regarding the likelihood, magnitude, and timing of recovery. Such interpretation holds a significant promise for improving the reliability and prognostic utility of connectivity strength, potentially empowering clinicians to design more targeted and effective interventions in line with the goals of precision psychiatry. Finally, because connectivity strength pertains to basic properties of complex systems, its phase-dependent predictive value is likely generalizable across multiple levels of analysis and disciplines, including psychology, neuroscience, and social sciences.

## Supporting information

10.1192/j.eurpsy.2025.10083.sm001 Delli Colli et al. supplementary material Delli et al. supplementary material

## Data Availability

The dataset used in the present study is available through the NIMH Data Archive. Researchers interested in using the Sequenced Treatment Alternatives to Relieve Depression (STAR*D) dataset are supposed to request it from the NIMH (https://nda.nih.gov/).

## Supplementary material

The supplementary material for this article can be found at http://doi.org/10.1192/j.eurpsy.2025.10083.

## Code availability

The R codes used in this study is publicly available at: https://github.com/clacollins/European-Psychiatry.

## References

[1] Branchi I. The double edged sword of neural plasticity: Increasing serotonin levels leads to both greater vulnerability to depression and improved capacity to recover. Psychoneuroendocrinology. 2011;36(3):339–51. 10.1016/j.psyneuen.2010.08.011. 20875703

[2] Branchi I. Plasticity in mental health: A network theory. Neurosci Biobehav Rev. 2022;138:104691 10.1016/j.neubiorev.2022.104691. 35568207

[3] Branchi I. Integrating plasticity into precision psychiatry. Eur Psychiatry. 2025;1–12. 10.1192/j.eurpsy.2025.19. PMC12041723 39935361

[4] Branchi I, Giuliani A. Shaping therapeutic trajectories in mental health: Instructive vs. permissive causality. Eur Neuropsychopharmacol. 2021;43:1–9. 10.1016/j.euroneuro.2020.12.001. 33384216

[5] Chiarotti F, Viglione A, Giuliani A, Branchi I. Citalopram amplifies the influence of living conditions on mood in depressed patients enrolled in the STAR*D study. Transl Psychiatry. 2017;7(3):e1066 10.1038/tp.2017.35. 28323288 PMC5416678

[6] Viglione A, Chiarotti F, Poggini S, Giuliani A, Branchi I. Predicting antidepressant treatment outcome based on socioeconomic status and citalopram dose. Pharmacogenomics J. 2019;19(6):538–46. 10.1038/s41397-019-0080-6. 30723316

[7] Cuijpers P, Karyotaki E, Ciharova M, Miguel C, Noma H, Furukawa TA. The effects of psychotherapies for depression on response, remission, reliable change, and deterioration: A meta-analysis. Acta Psychiatry Scand. 2021;144(3):288–99. 10.1111/acps.13335. PMC8457213 34107050

[8] Delli Colli C, Viglione A, Poggini S, Cirulli F, Chiarotti F, Giuliani A, et al. A network-based analysis anticipates time to recovery from major depression revealing a plasticity by context interplay. Transl Psychiatry. 2025;15(1):32 10.1038/s41398-025-03246-1. 39875363 PMC11775195

[9] Bottemanne H, Morlaas O, Claret A, Sharot T, Fossati P, Schmidt L. Evaluation of early ketamine effects on belief-updating biases in patients with treatment-resistant depression. JAMA Psychiatry. 2022; 10.1001/jamapsychiatry.2022.2996. PMC9520441 36169969

[10] Carhart-Harris RL, Roseman L, Haijen E, Erritzoe D, Watts R, Branchi I, et al. Psychedelics and the essential importance of context. J Psychopharmacol. 2018;32(7):725–31. 10.1177/0269881118754710. 29446697

[11] Klobl M, Seiger R, Vanicek T, Handschuh P, Reed MB, Spurny-Dworak B, et al. Escitalopram modulates learning content-specific neuroplasticity of functional brain networks. NeuroImage. 2022;247:118829 10.1016/j.neuroimage.2021.118829. 34923134

[12] Belsky J, Jonassaint C, Pluess M, Stanton M, Brummett B, Williams R. Vulnerability genes or plasticity genes? Mol Psychiatry. 2009;14(8):746–54. 10.1038/mp.2009.44. 19455150 PMC2834322

[13] Borsboom D, Cramer AO. Network analysis: An integrative approach to the structure of psychopathology. Annu Rev Clin Psychol. 2013;9:91–121. 10.1146/annurev-clinpsy-050212-185608. 23537483

[14] Borsboom D. A network theory of mental disorders. World Psychiatry. 2017;16(1):5–13. 10.1002/wps.20375. 28127906 PMC5269502

[15] Scheffer M, Bockting CL, Borsboom D, Cools R, Delecroix C, Hartmann JA, et al. A dynamical systems view of psychiatric disorders-theory: A review. JAMA Psychiatry. 2024;81(6):618–23. 10.1001/jamapsychiatry.2024.0215. 38568615

[16] Branchi I. A mathematical formula of plasticity: Measuring susceptibility to change in mental health and data science. Neurosci Biobehav Rev. 2023;152:105272 10.1016/j.neubiorev.2023.105272. 37277011

[17] Delli Colli C, Chiarotti, F., Campolongo, P., Giuliani, A., Branchi I. Towards a network-based operationalization of plasticity for predicting the transition from depression to mental health. Nat Mental Health 2024;2(February 2024):200–8. 10.1038/s44220-023-00192-z.

[18] Viglione A, Delli Colli C, Poggini S, Mobasher M, Vai B, Poletti S, et al. A network-based approach reveals higher plasticity levels in bipolar than major depressive disorder. J Affect Disord. 2025;387:119549 10.1016/j.jad.2025.119549. 40441646

[19] Ashaie SA, Hung J, Funkhouser CJ, Shankman SA, Cherney LR. Depression over time in persons with stroke: A network analysis approach. J Affect Disord Rep. 2021;4 10.1016/j.jadr.2021.100131. PMC8438599 34528021

[20] Lee CT, Kelley SW, Palacios J, Richards D, Gillan CM. Estimating the prognostic value of cross-sectional network connectivity for treatment response in depression. Psychol Med. 2024;54(2):317–26. 10.1017/S0033291723001368. 37282838

[21] van Borkulo C, Boschloo L, Borsboom D, Penninx BW, Waldorp LJ, Schoevers RA. Association of Symptom Network Structure with the course of [corrected] depression. JAMA Psychiatry. 2015;72(12):1219–26. 10.1001/jamapsychiatry.2015.2079. 26561400

[22] McElroy E, Napoleone E, Wolpert M, Patalay P. Structure and connectivity of depressive symptom networks corresponding to early treatment response. EClinicalMedicine. 2019;8:29–36. 10.1016/j.eclinm.2019.02.009. 31193604 PMC6537518

[23] Lorimer B, Delgadillo J, Kellett S, Brown G. Exploring relapse through a network analysis of residual depression and anxiety symptoms after cognitive behavioural therapy: A proof-of-concept study. Psychother Res. 2020;30(5):650–61. 10.1080/10503307.2019.1650980. 31382844

[24] O’Driscoll C, Buckman JEJ, Fried EI, Saunders R, Cohen ZD, Ambler G, et al. The importance of transdiagnostic symptom level assessment to understanding prognosis for depressed adults: Analysis of data from six randomised control trials. BMC Med. 2021;19(1):109 10.1186/s12916-021-01971-0. 33952286 PMC8101158

[25] Schweren L, van Borkulo CD, Fried E, Goodyer IM. Assessment of symptom network density as a prognostic marker of treatment response in adolescent depression. JAMA Psychiatry. 2018;75(1):98–100. 10.1001/jamapsychiatry.2017.3561. 29188277 PMC5833532

[26] Haken H. Advanced Synergetics: Instability hierarchies of self-organizing systems and devices. Springer; 1983.

[27] Rush AJ, Trivedi MH, Wisniewski SR, Nierenberg AA, Stewart JW, Warden D, et al. Acute and longer-term outcomes in depressed outpatients requiring one or several treatment steps: A STAR*D report. Am J Psychiatry 2006;163(11):1905–1917. 10.1176/ajp.2006.163.11.1905. 17074942

[28] Trivedi MH, Rush AJ, Wisniewski SR, Nierenberg AA, Warden D, Ritz L, et al. Evaluation of outcomes with citalopram for depression using measurement-based care in STAR*D: Implications for clinical practice. Am J Psychiatry 2006;163(1):28–40. 10.1176/appi.ajp.163.1.28. 16390886

[29] Burger J, Isvoranu AM, Lunansky G, Haslbeck JMB, Epskamp S, Hoekstra RHA, et al. Reporting standards for psychological network analyses in cross-sectional data. Psychol Methods. 2023;28(4):806–24. 10.1037/met0000471. 35404629

[30] van Borkulo CD, van Bork R, Boschloo L, Kossakowski JJ, Tio P, Schoevers RA, et al. Comparing network structures on three aspects: A permutation test. Psychol Methods. 2023;28(6):1273–85. 10.1037/met0000476. 35404628

[31] Epskamp S, Fried EI. A tutorial on regularized partial correlation networks. Psychol Methods. 2018;23(4):617–34. 10.1037/met0000167. 29595293

[32] Berlim MT, Richard-Devantoy S, Dos Santos NR, Turecki G. The network structure of core depressive symptom-domains in major depressive disorder following antidepressant treatment: A randomized clinical trial. Psychol Med. 2021;51(14):2399–413. 10.1017/S0033291720001002. 32312344

[33] Beard C, Millner AJ, Forgeard MJ, Fried EI, Hsu KJ, Treadway MT, et al. Network analysis of depression and anxiety symptom relationships in a psychiatric sample. Psychol Med. 2016;46(16):3359–69. 10.1017/S0033291716002300. 27623748 PMC5430082

[34] Bos FM, Fried EI, Hollon SD, Bringmann LF, Dimidjian S, DeRubeis RJ, et al. Cross-sectional networks of depressive symptoms before and after antidepressant medication treatment. Soc Psychiatry Psychiatr Epidemiol. 2018;53(6):617–27. 10.1007/s00127-018-1506-1. 29627898 PMC5959987

[35] Schumacher L, Klein JP, Elsaesser M, Harter M, Hautzinger M, Schramm E, et al. Implications of the network theory for the treatment of mental disorders: A secondary analysis of a randomized clinical trial. JAMA Psychiatry. 2023;80(11):1160–8. 10.1001/jamapsychiatry.2023.2823. 37610747 PMC10448377

[36] Blanken TF, Van Der Zweerde T, Van Straten A, Van Someren EJW, Borsboom D, Lancee J. Introducing network intervention analysis to investigate sequential, symptom-specific treatment effects: A demonstration in co-occurring insomnia and depression. Psychother Psychosom. 2019;88(1):52–4. 10.1159/000495045. 30625483 PMC6469840

[37] Lunansky G, Hoekstra RHA, Blanken TF. Disentangling the role of affect in the evolution of depressive complaints using complex dynamical networks. Collabra: Psychology. 2023;9(1). 10.1525/collabra.74841.

[38] Kelley SW, Fisher AJ, Lee CT, Gallagher E, Hanlon AK, Robertson IH, et al. Elevated emotion network connectivity is associated with fluctuations in depression. Proc Natl Acad Sci USA. 2023;120(45):e2216499120 10.1073/pnas.2216499120. 37903279 PMC10636367

[39] Terluin B, de Boer MR, de Vet HC. Differences in connection strength between mental symptoms might be explained by differences in variance: Reanalysis of network data did not confirm staging. PLoS One. 2016;11(11):e0155205 10.1371/journal.pone.0155205. 27880771 PMC5120783

[40] Scheffer M, Bascompte J, Brock WA, Brovkin V, Carpenter SR, Dakos V, et al. Early-warning signals for critical transitions. Nature. 2009;461(7260):53–9. 10.1038/nature08227. 19727193

[41] van de Leemput IA, Wichers M, Cramer AO, Borsboom D, Tuerlinckx F, Kuppens P, et al. Critical slowing down as early warning for the onset and termination of depression. Proc Natl Acad Sci USA 2014;111(1):87–92. 10.1073/pnas.1312114110. 24324144 PMC3890822

[42] Wichers M, Smit AC, Snippe E. Early warning signals based on momentary affect dynamics can expose nearby transitions in depression: A confirmatory single-subject time-series study. J Pers Oriented Res. 2020;6(1):1–15. 10.17505/jpor.2020.22042. 33569148 PMC7842626

[43] Koenders MA, de Kleijn R, Giltay EJ, Elzinga BM, Spinhoven P, Spijker AT. A network approach to bipolar symptomatology in patients with different course types. PLoS One. 2015;10(10):e0141420 10.1371/journal.pone.0141420. 26505477 PMC4624774

[44] Bury TM, Sujith RI, Pavithran I, Scheffer M, Lenton TM, Anand M, et al. Deep learning for early warning signals of tipping points. Proc Natl Acad Sci USA. 2021;118(39): 10.1073/pnas.2106140118. PMC8488604 34544867

[45] Kefi S, Guttal V, Brock WA, Carpenter SR, Ellison AM, Livina VN, et al. Early warning signals of ecological transitions: Methods for spatial patterns. PLoS One. 2014;9(3):e92097 10.1371/journal.pone.0092097. 24658137 PMC3962379

[46] de Felice G, Giuliani A, Pincus D, Scozzari A, Berardi V, Kratzer L, et al. Stability and flexibility in psychotherapy process predict outcome. Acta Psychol 2022;227:103604. 10.1016/j.actpsy.2022.103604. 35537234

[47] Gorban AN, Smirnova EV, Tyukina TA. Correlations, risk and crisis: From physiology to finance. Phys A. 2010;389(16):3193–217. 10.1016/j.physa.2010.03.035.

[48] Lee Pe M, Kircanski K, Thompson RJ, Bringmann LF, Tuerlinckx F, Mestdagh M, et al. Emotion-network density in major depressive disorder. Clin Psychol Sci. 2015;3(2):292–300. 10.1177/2167702614540645. 31754552 PMC6871506

[49] Kelley SW, Gillan CM. Using language in social media posts to study the network dynamics of depression longitudinally. Nat Commun. 2022;13(1):870 10.1038/s41467-022-28513-3. 35169166 PMC8847554

[50] Kraus C, Kadriu B, Lanzenberger R, Zarate Jr. CA, Kasper S. Prognosis and improved outcomes in major depression: A review. Focus. 2020;18(2):220–35. 10.1176/appi.focus.18205. PMC7587891 33343240

[51] Rost N, Binder EB, Bruckl TM. Predicting treatment outcome in depression: An introduction into current concepts and challenges. Eur Arch Psychiatry Clin Neurosci. 2023;273(1):113–27. 10.1007/s00406-022-01418-4. 35587279 PMC9957888

[52] Bergfeld IO, Mantione M, Figee M, Schuurman PR, Lok A, Denys D. Treatment-resistant depression and suicidality. J Affect Disord. 2018;235:362–7. 10.1016/j.jad.2018.04.016. 29665520

[53] Penninx BW, Nolen WA, Lamers F, Zitman FG, Smit JH, Spinhoven P, et al. Two-year course of depressive and anxiety disorders: Results from the Netherlands study of depression and anxiety (NESDA). J Affect Disord. 2011;133(1–2):76–85. 10.1016/j.jad.2011.03.027. 21496929

[54] Kohler S, Chrysanthou S, Guhn A, Sterzer P. Differences between chronic and nonchronic depression: Systematic review and implications for treatment. Depress Anxiety. 2019;36(1):18–30. 10.1002/da.22835. 30300454

[55] Lundberg J, Cars T, Loov SA, Soderling J, Sundstrom J, Tiihonen J, et al. Association of Treatment-Resistant Depression with Patient Outcomes and Health Care Resource Utilization in a population-wide study. JAMA Psychiatry. 2023;80(2):167–75. 10.1001/jamapsychiatry.2022.3860. 36515938 PMC9856735

[56] Skelton M, Carr E, Buckman JEJ, Davies MR, Goldsmith KA, Hirsch CR, et al. Trajectories of depression and anxiety symptom severity during psychological therapy for common mental health problems. Psychol Med. 2023;53(13):6183–93. 10.1017/S0033291722003403. 36510471 PMC10520600

