# Supplementary material for: Anticipating depression trajectories by measuring plasticity and change through symptom network dynamics
Source: Eur Psychiatry. 2025 Aug 15;68(1):e128. doi: 10.1192/j.eurpsy.2025.10083 (PMC12438987; doi:10.1192/j.eurpsy.2025.10083)
Supplement: Delli Colli et al. supplementary material — Delli et al. supplementary material [file S0924933825100837sup001.docx]

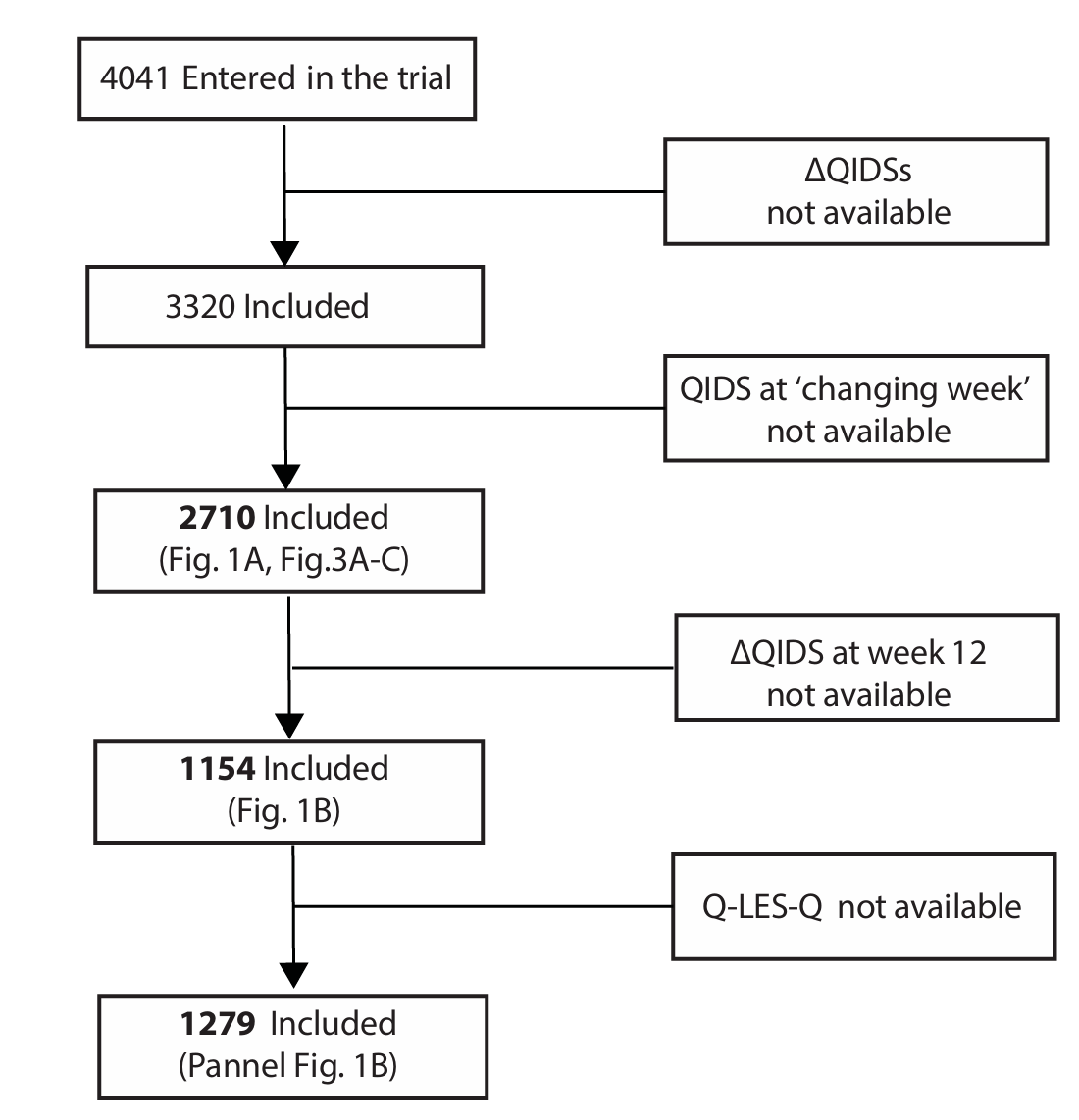


**Figure S1.** Flow chart showing the patients included and the corresponding analyses in which each sample was used. Q-LES-Q-SF, Quality of Life Enjoyment and Satisfaction Questionnaire – Short Form. QIDS, Quick Inventory of Depressive Symptomatology.


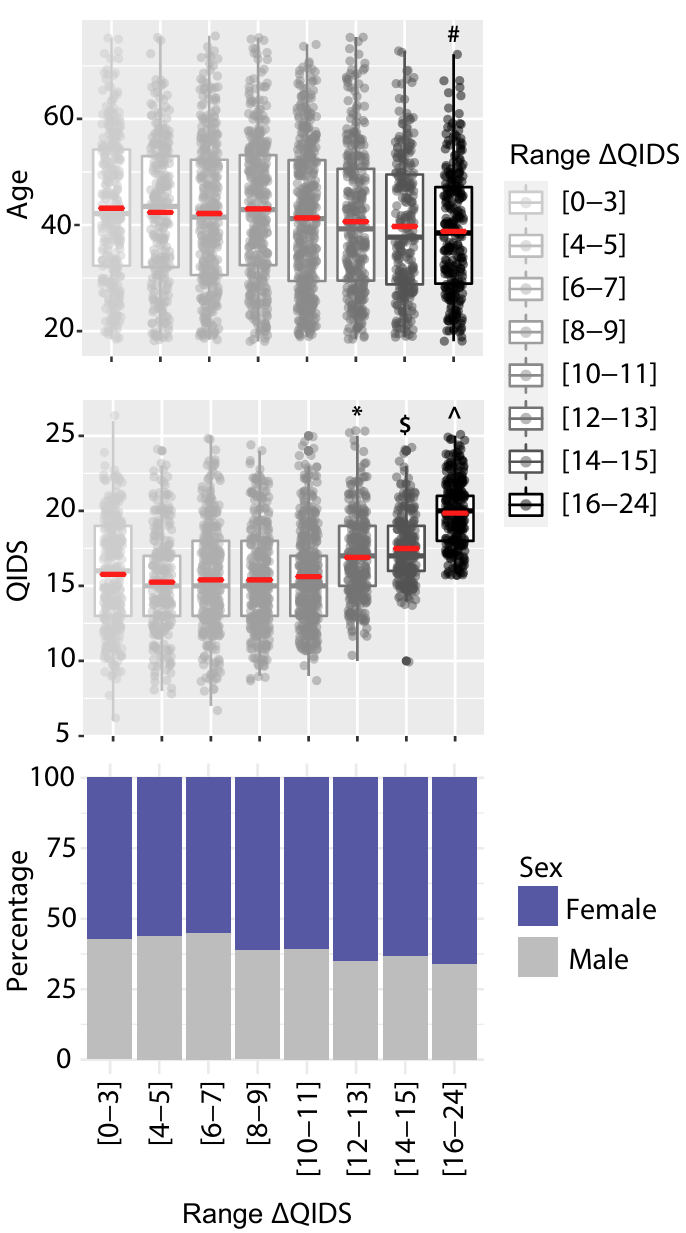


**Figure S2. Baseline sample characteristics.** Post-hoc comparisons performed using the Tukey test. #, [16-24] *vs.* [8-9], [6-7], [4-5],[0-3]; ^[16-24] *vs*. all; $, [14-15] vs. all; *[12-13] *vs.* all. #,$,* and ^p-value < 0.001.


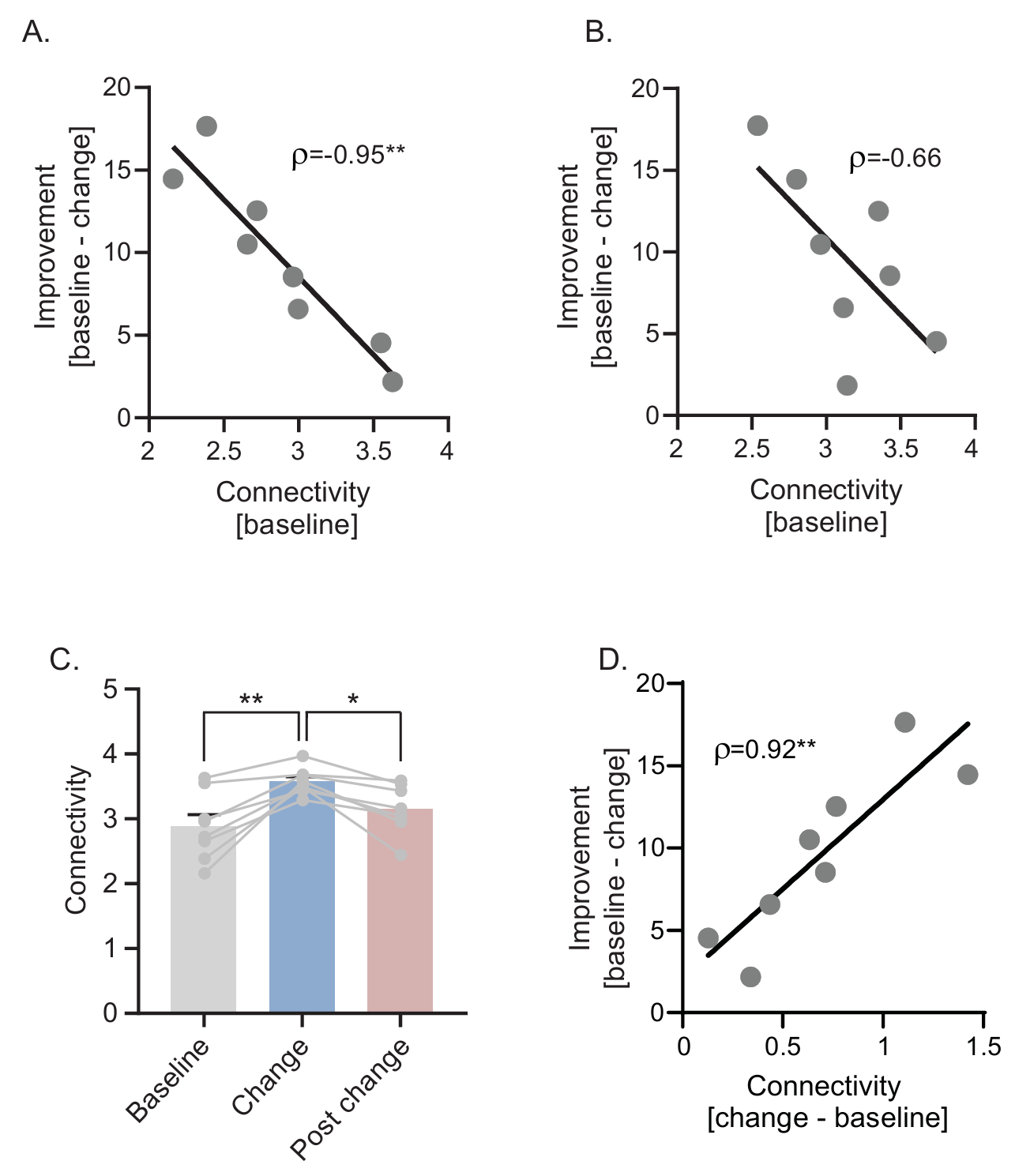


**Figure S2. Analysis included only subjects who showed clinical improvement over time.** Connectivity strength was inversely correlated with (A) the maximum clinical change achieved across the study period and (B) the change observed by week 12. (C) Changes in connectivity strength predicted the maximum clinical improvement. Connectivity strength significantly increased from baseline during the change phase. Two-tailed paired *t*-tests with Bonferroni correction were used: *p* = 0.02, p = 0.007. (D) Correlation between the change in connectivity strength (from baseline to the change phase) and the maximum clinical improvement, expressed as ΔQIDS averaged within each group. A two-sided Spearman rank correlation test was used; ρ represents the Spearman coefficient. p < 0.01.
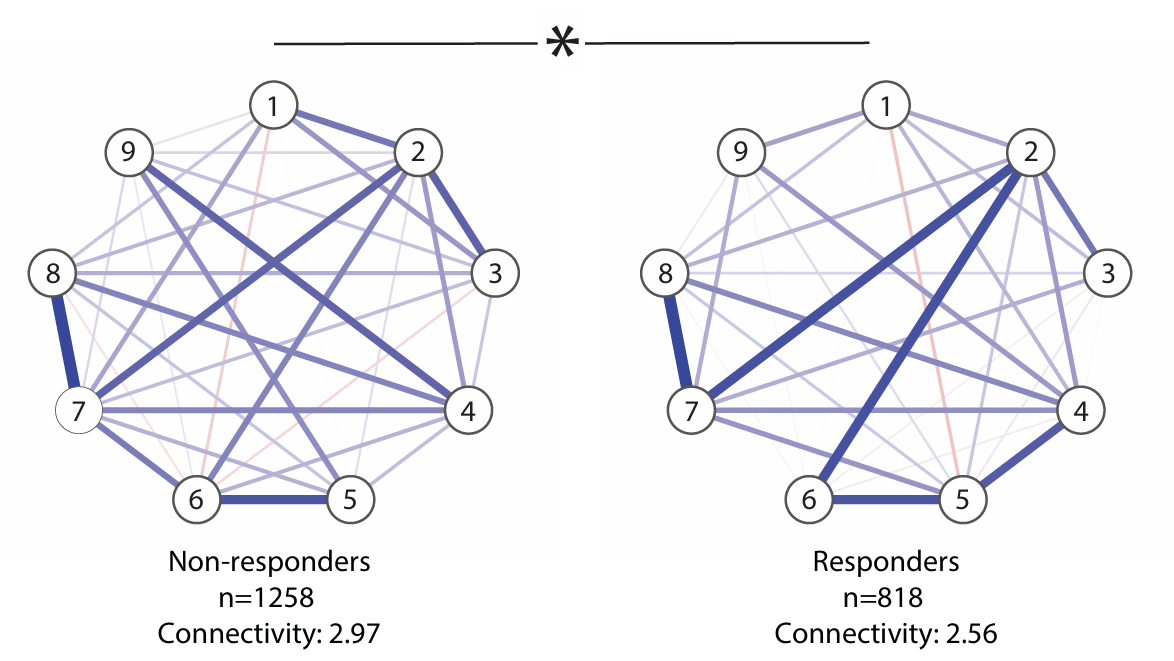


**Figure S3. Connectivity strength** **in subjects who consistently achieved clinical response at any of the available weeks to those who did not**. Blue connections represent positive associations, whereas red connections represent negative associations. Thicker edges represent stronger associations (both for positive and negative). 1 - sleeping problem (sleep onset insomnia, mid, early and hypersomnia); 2 - depressed mood; 3 - weight/appetite change; 4 - concentration/decision making; 5 - outlook (self); 6 - suicidal ideation; 7 - involvement; 8 - energy/fatigability; 9 - psychomotor agitation. Network comparison test (NCT) was used to compare groups. *p<0.05.


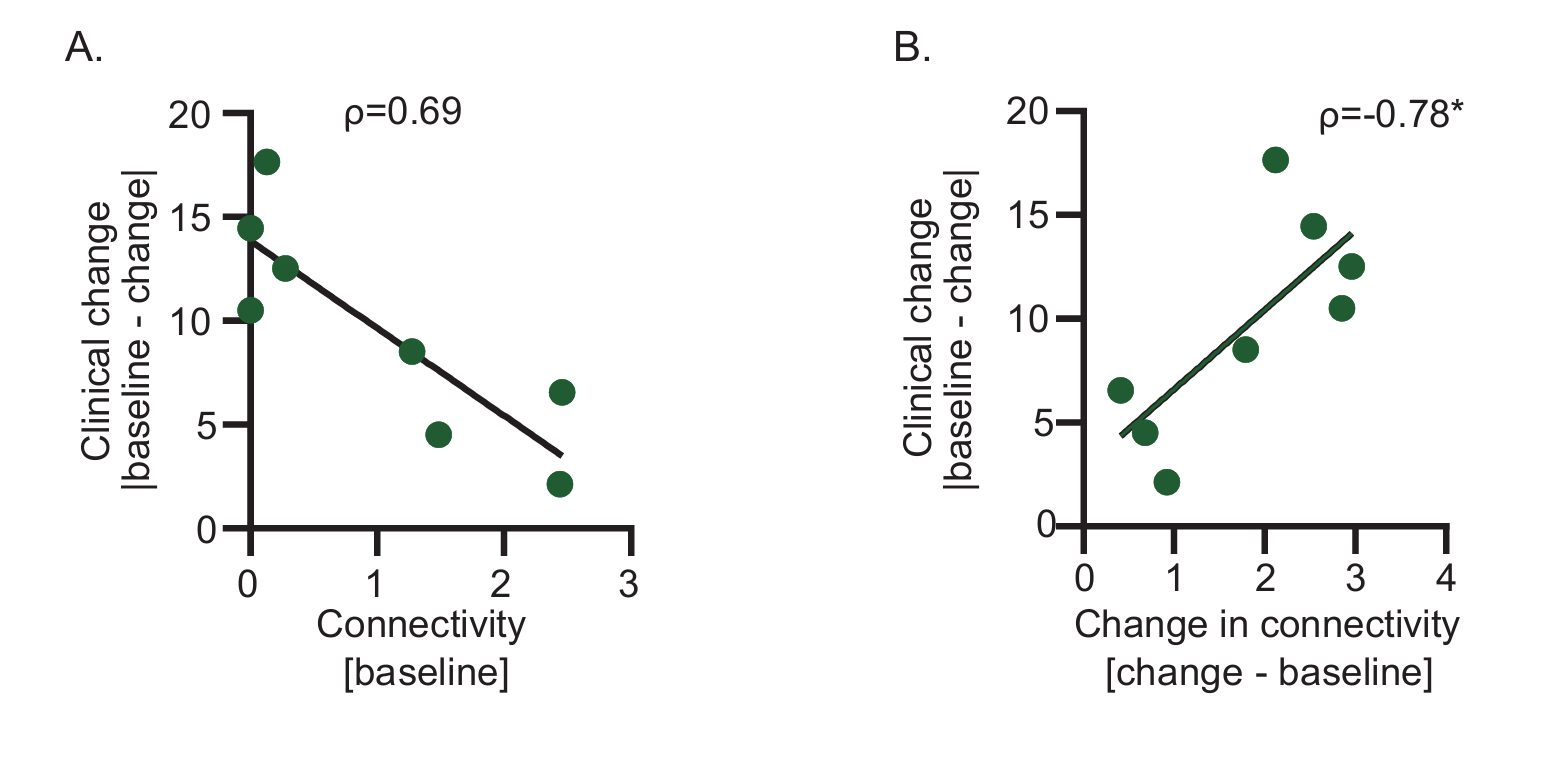


**Figure S4. Replication of the analysis Fig. 1A and Fig. 2B by estimating the network with regularization.** A. Correlation between baseline connectivity and maximum change. B. Correlation between change in connectivity and maximum change. Maximum modification, mean ∆QIDS calculated from baseline to week of maximum modification. A two-sided Spearman rank correlation test was used to estimate the correlation. ρ, spearman coefficient.


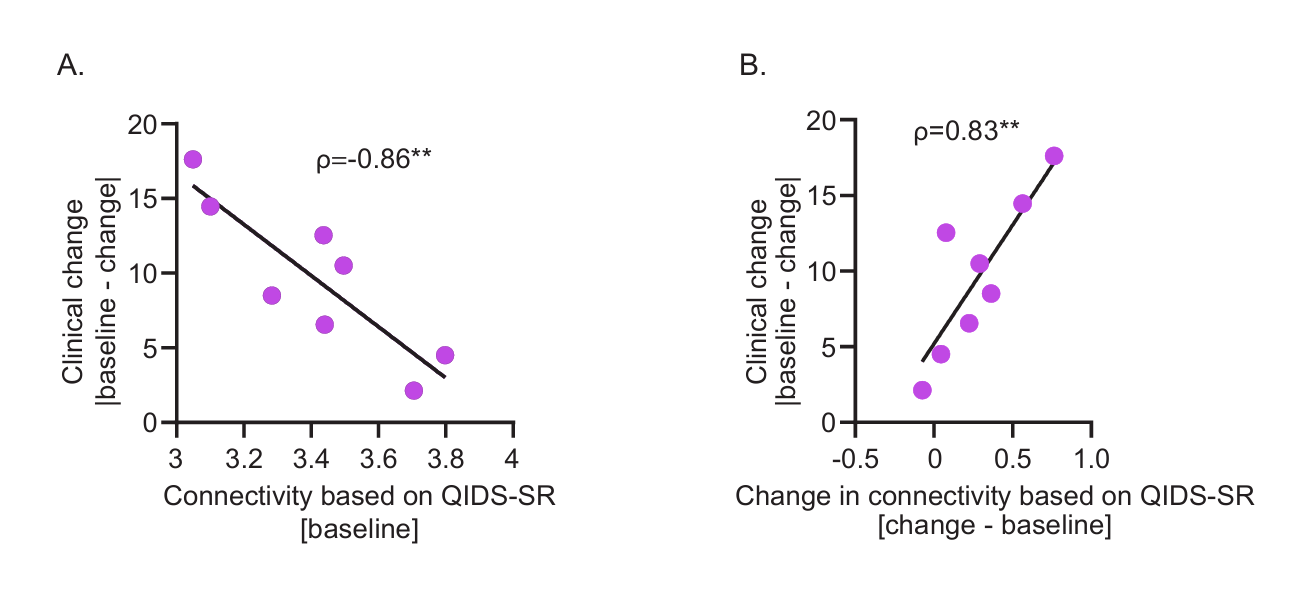


**Figure S5. Replication of the analysis Fig. 1A and Fig. 2B by estimating the connectivity strength at baseline using the self-reported QIDS version (QIDS-SR)** A. Correlation between baseline connectivity and maximum modification. B. Correlation between change in connectivity and maximum change. Maximum modification, mean ∆QIDS calculated from baseline to week of maximum modification. A two-sided Spearman rank correlation test was used to estimate the correlation. ρ, spearman coefficient.


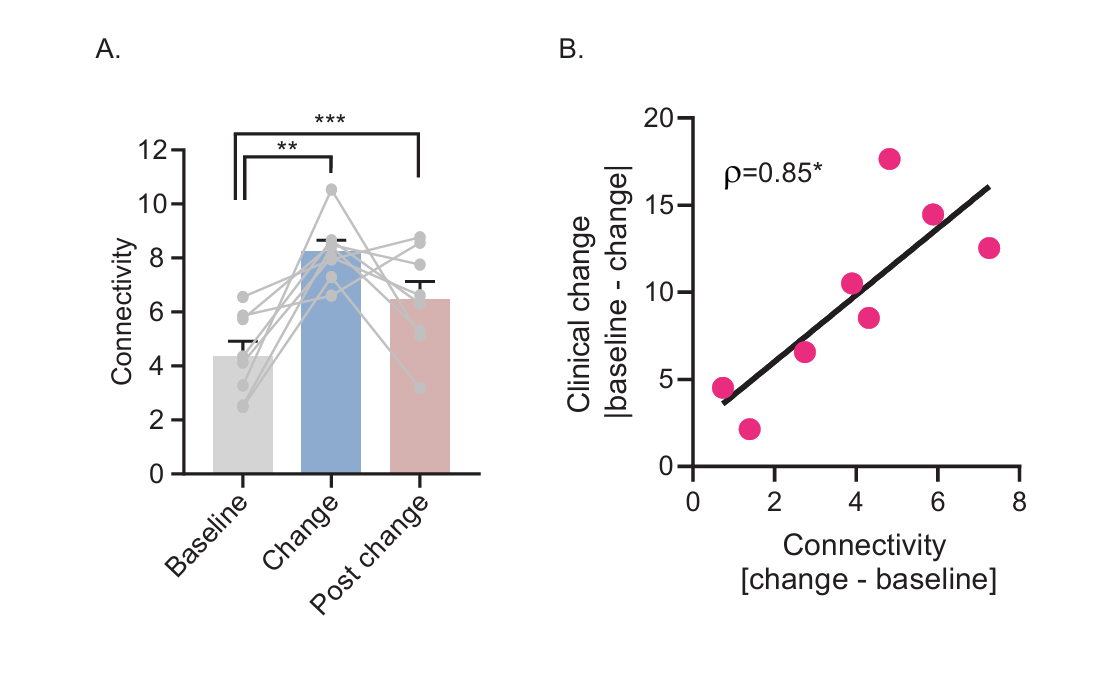


**Figure S6. Network estimation using full correlation**. Change in connectivity strength predicts the maximum clinical change achieved across the weeks. (A) Connectivity strength increases from baseline during the change phase. Two-tailed paired t-tests with Bonferroni correction: **p-value<0.01, ***p-value<0.001. (B) Correlation between the change in connectivity strength from baseline to change phase and the maximum clinical change (i.e., ΔQIDS averaged within each group). A two-sided Spearman rank correlation test was used to estimate the correlation. ρ, spearman coefficient, *p-value=0.01.**Table 1**

| Baseline [week] | Maximum clinical change [week] | Change phase [week] | Post-change phase [week] |
| --- | --- | --- | --- |
| 0 | 4 | 2 | 4 |
| 0 | 6 | 4 | 6 |
| 0 | 9 | 6 | 9 |
| 0 | 12 | 9 | 12 |
